# Supplementary material for: Modeling Emotional Arousal With Turbulence Measured by EEG
Source: Psychophysiology. 2025 Jun 20;62(6):e70093. doi: 10.1111/psyp.70093 (PMC12181742; doi:10.1111/psyp.70093)
Supplement: Supplementary file 1 — Data S1. [file PSYP-62-e70093-s001.pdf]

Received: Added at production | Revised: Added at production | Accepted: Added at production
DOI: xxx/xxxx

ARTICLE TYPE

Modeling emotional arousal with turbulence measured by EEG

Marc Vidal[1,2,3] | Nádia Moura[4] | Bavo Van Kerrebroeck[1,5] | Ana M. Aguilera[2] | Thomas H. Fritz[3,1] | Marc Leman[1]

[1]IPEM and Department of Data Analysis, University of Ghent, B-9000 Ghent, Belgium
[2]Department of Statistics and Institute of Mathematics, University of Granada, 18071 Granada, Spain
[3]Department of Neurology, Max Planck Institute for Human Cognitive and Brain Sciences, 04103 Leipzig, Germany
[4]University of Coimbra, CEIS20, Faculty of Arts and Humanities, Coimbra, Portugal
[5]Department of Psychology and Department of Music Research, CIRMMT, McGill University

Correspondence
Marc Vidal and Thomas H. Fritz
Email: marc.vidalbadia@ugent.be (M.V.); fritz@cbs.mpg.de (T.H.F.)

Funding Information
This research was supported by the Methusalem funding from the Flemish Government and the project FQM-307 of the Government of Andalusia (Spain). We also acknowledge the financial support of Agencia Estatal de Investigación, Ministerio de Ciencia e Innovación (grant number: PID2020-113961GB-I00) and the IMAG María de Maeztu grant CEX2020-001105.

Abstract

Turbulence-like dynamics in brain activity have been proposed as a signature of systems operating near criticality, and may reflect changes in neuronal function associated with emotional states. In this paper, we hypothesize that motor behavior linked to emotional expression modulates turbulence, reflecting a shift toward more streamlined brain dynamics characteristic of emotional motor control. We assessed EEG turbulence in 30 healthy participants in a motor paradigm varying in both task demand and degree of emotionality. Conditions included singing, swaying, responding to a virtual conductor of variable expressivity, having your own body movements mirrored by a virtual agent, and combinations thereof. Results showed an inverse relation of turbulence intensity in the alpha range to both degree of movement and perceived level of task emotionality, which was also true for the high gamma range, but to a lesser extent. When factoring in task demand, the effect of level of emotionality in the alpha range deteriorated. This is physiological evidence for why physical arousal is likely to increase the level of perceived emotional engagement or even be misinterpreted as such. Our findings suggest high gamma activity is a more accurate indicator of emotionality during motor tasks and can be key to differentiating EEG signatures of emotional motor control, which has been shown to be partly autonomous from voluntary motor control.

Impact Statement: Emotional arousal and motor engagement are tightly intertwined during expressive behaviors like singing. Here, we introduce an EEG-based method to quantify turbulence-like neural dynamics and assess how they reflect emotional involvement during motor performance. By analyzing alpha and high gamma activity across experimental conditions varying in motor execution and task expressivity, we show that turbulence intensity is inversely related to emotional engagement and motor control—particularly in high gamma. These findings offer new insights for investigating emotion-related brain dynamics in naturalistic, embodied tasks, with potential relevance for both fundamental research in motor control and interventions targeting emotional and volitional motor impairments.

KEYWORDS
criticality, emotional motor control, functional data, immersive VR, mind-brain-body, multisensory processing, naturalistic neuroscience

1 | INTRODUCTION

Singing is one of the most archaic and refined forms of human emotional expression. From an evolutionary perspective, the intimate relationship between singing and emotion can be explained by the adaptive functions of music (Gray et al. 2001, Fitch 2005), ranging from mating selection (Iwasaki et al. 2013, Herman 2017) to social bonding (Sasaki et al. 2006, Weinstein et al. 2016, Bowling et al. 2022) or caregiving (Cirelli et al. 2019, Jover et al. 2019, Lense et al. 2022). Its neural basis is closely tied to the emotional motor system, with vocal production involving intricate coordination between brainstem regions, such as the nucleus retroambiguus and cortical areas (Holstege et al. 1996, Holstege and Subramanian 2015). Two complementary neural pathways facilitate this process: one linking the anterior cingulate cortex and midbrain periaqueductal gray to voluntary initiation and emotional control, and the other connecting the primary motor cortex with subcortical circuits that fine-tune motor commands (Jürgens 2009, Owren et al. 2011). Singing (or listening to human singing) also involves specific brain areas such as the insula and parietal regions, anterior superior temporal

 © 2025 Copyright Holder Name

gyrus, among others, influencing interactions with networks of selective neural populations in sensory-motor areas beyond the auditory cortex (Kleber et al. 2010 2013, Zarate 2013, Lévêque and Schön 2015, Staib and Frühholz 2021, Norman-Haignere et al. 2022). Research in humans and non-human animals suggests that brainstem neurotransmitters, and particularly acetylcholine, play a significant role in shaping singing behavior by modulating motor control and emotional responses (Sasaki et al. 2006, Jaffe and Brainard 2020, Gritton et al. 2024, Vidal et al. 2024). These underlying distributed neural mechanisms highlight singing's unique capacity to unify cognitive, affective, and motor processes, making it an appropriate paradigm for the study of neurological signatures of emotional engagement and motor control.

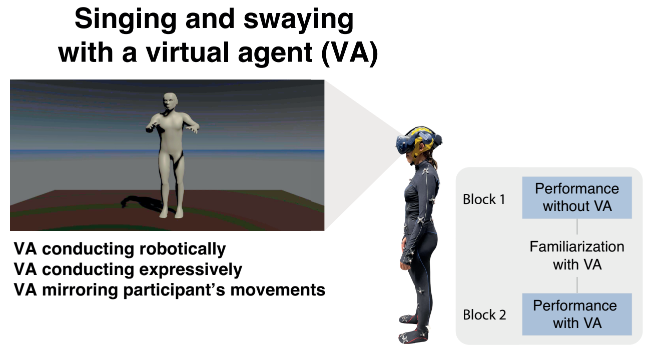

FIGURE 1 Experimental paradigm. Participants engaged in a set of conditions combining singing, swaying and interacting with three virtual agents conducting music (robotically, expressively and mirroring participants' movements), while wearing a mocap suit, VR headset, and EEG cap. The experiment was organized in two blocks of randomized conditions, without and with VA. A short familiarization task with the VA took place before the VA block.

Here, we investigate how active participation in musical tasks, including singing and moving along to the music, modulates emotional engagement and cortical electrophysiological responses. We hypothesize that active engagement in such tasks will elicit greater emotional involvement than passive conditions, providing a framework to delve into the neurology of emotion and emotional motor control. In this study, we incorporate virtual reality as a novel means to explore the social dimension underlying music experience, ensuring an environment that closely mimics real-world conditions while allowing for the use of complex physiological apparatus (see Fig.1). To systematically investigate the interplay between emotional engagement, motor activity, and social interaction, we experimentally introduce three different task-specific conditions: (1) singing (singing vs. non-singing), (2) moving (moving vs. non-moving), and (3) virtual agent (VA) interaction (expressive vs. robotic vs. mirroring vs. no VA). These dimensions were selected to manipulate emotional engagement by varying the level of motor involvement and interaction, allowing us to isolate their individual and combined effects. The previous conditions produce six different tasks: without VA, non-movement singing (NM.S.NA) and movement singing (M.S.NA), and with VA, movement non-singing mirroring VA (M.NS.Mir), movement singing mirroring VA (M.S.Mir), non-movement singing robotic VA (NM.S.Rob), and non-movement singing expressive VA (NM.S.Exp).

Although the dual movement paradigm has been widely adopted in music research (Bangert and Altenmüller 2003, Koelsch et al. 2006, Moura et al. 2024, Spiech et al. 2024, Vidal et al. 2024, to cite a few), its inclusion in the study of emotional arousal using electroencephalography (EEG) is rarely observed. Some studies on alpha/mu rhythms, typically in the 8-13 Hz range, found that increased power is associated with inhibition of action during music listening (Ross et al. 2021), while reduced power is likely linked to cognitive demand (Ehinger et al. 2014), motor function (Arroyo et al. 1993, Ross et al. 2021), and possibly emotional arousal, particularly on parieto-occipital electrodes (see Hofmann et al. (2020) and references therein). However, it remains unclear to what extent these effects are specific to emotional arousal or are related to movement-induced arousal. Hofmann et al. (2020), who also adopted a motor paradigm with VR, suggested that higher frequencies could provide additional information on these processes, potentially improving model predictions.

In this paper, we concentrate on alpha (8-13 Hz) and high gamma (50-80 Hz) frequency bands to build on and extend previous findings. The choice of high gamma is motivated in part by the fact that the 30–50 Hz range is particularly vulnerable to motor and movement-related artifacts, including electromyographic activity from facial and neck muscles (Whitham et al. 2007). This lower gamma band is also more susceptible to broadband, non-oscillatory signals linked to motor preparation and cortical desynchronization (Cheyne et al. 2008, Muthukumaraswamy 2010). In contrast, the 50–80 Hz sub-band captures narrow-band gamma oscillations that are less prone to these confounds and more functionally specific, particularly during emotional states (Yang et al. 2020). These oscillations are selectively enhanced by predictable sensory input and have been proposed to support cortical stabilization and feedback integration (Vinck et al. 2025, Bartoli et al. 2019). While broader affective–brain interplay has been demonstrated through studies of central–autonomic coupling (Calderon et al. 2016, Candia-Rivera et al. 2022, Fourcade et al. 2024), direct EEG evidence linking high gamma activity to emotional state distinctions is still emerging (Yang et al. 2020), and its investigation during overt movement remains relatively uncommon. To meet the associated data quality demands, we used advanced Wavelet methods to prevent, identify, and address artifacts in the EEG signal (see 3.6 and 3.7 for details). Furthermore, a unique aspect of our study in relation to the previous one is the tracking of bodily movement with motion capture technology, enabling us to incorporate quantitative motor measures in our analyses.

The present paradigm investigates whether alpha and high gamma responses reflect distinct physiological signatures of motor and emotional arousal. As detailed in the next section, we introduce a turbulence-based EEG metric to characterize how neural dynamics unfold across varying levels of movement, emotional engagement, and interaction

with a virtual agent. This allows us to test whether emotional motor control is associated with frequency-specific changes in turbulence, potentially reflecting more regulated or parsimonious neural states under emotional and motor demands.

2 | MODELING GENERALIZED CNS AROUSAL THROUGH EEG TURBULENCE

The relevance of brainstem function to singing brings forth the notion of generalized CNS arousal (GA) (Pfaff 2009, Pfaff et al. 2012, Martin and Pfaff 2013, Calderon et al. 2016, Kilinc et al. 2023). GA is considered the composite result of multiple neuromodulatory systems operating at a high hierarchical level in the brain, commonly manifested through behavioral activation. This form of arousal has been widely investigated using electrophysiological measures (Hudson et al. 2014, Calderon et al. 2016, Gao et al. 2019, Ribeiro et al. 2022). While existing studies have proposed several quantitative descriptors for GA (Quinkert et al. 2011, Pfaff et al. 2012, Proekt et al. 2012, Calderon et al. 2016, Vidal et al. 2024), often focusing on dominant component estimation, our work introduces a Hilbertian framework for examining turbulent dynamics in the EEG signal. Given that postsynaptic potentials are influenced by neurotransmitter release at axonal terminals — and that neuromodulatory activity is spatially diffuse across the cortex (Ballinger et al. 2016) — turbulence in EEG signals may offer an indirect, integrative marker of GA function. In this context, turbulence refers to irregular, broadband fluctuations in EEG activity, marked by transient desynchronization of phase and amplitude across spatially distributed cortical regions. These dynamics exhibit scale-invariant structure, arising from nonlinear interactions among neural populations coordinated across multiple spatiotemporal scales (Deco et al. 2025). Here, we examine this property as a potential organizing principle of motor-related emotional arousal.

In recent years, differential equation modeling using ensembles of Stuart-Landau (Hopf) oscillators has been applied to characterize turbulence in functional magnetic resonance (fMRI) and magnetoencephalography (MEG) datasets (Deco and Kringelbach 2020, Escrichs et al. 2022, Deco et al. 2023), with limited exploration in the context of EEG studies. These approaches, however, provide a compact interpretable model in terms of the relationship between derivatives, while other reduction techniques achieve more effective dimension reduction from a geometric point of view (Berkooz et al. 1993, Aguilera et al. 1997, Rosa et al. 2014). Better insights into sources of variability can be further enhanced through multivariate considerations, encompassing models that factor in multiple dimensions, including time, space, and experimental conditions altogether, facilitating precise characterization of dynamic interdependencies inherent to neuroscientific data.

Here, we work under the paradigm of second-generation functional data (Koner and Staicu 2023). In the analysis of these data, typically assumed to belong to an infinite-dimensional Hilbert space, complex dependencies between functional observations are considered. Given the non-Gaussian nature of turbulence, we propose a functional independent component analysis (ICA) (Epifanio and Ventura-Campos 2014, Vidal et al. 2021 2025) extended to multivariate functional data that vary in spatial and temporal location to analyze turbulent flows in EEG brain activity. To uncover the presence of turbulent-like dynamics, this study performs spectral analysis of a kurtosis kernel function (i.e., the integral kernel of a kurtosis operator), examining its temporal effects on spatially projected data. We entirely work in the frequency domain, namely on the space of coefficients of the Hilbertian random element in the eigenbasis of the kurtosis function. By using overlapping windows to estimate these coefficients over time, we achieve a regularizing effect similar to the Welch method, which is known for its statistical consistency in power spectral density estimation. Our approach captures smooth spatial transitions through temporal dynamical modes, generating a vorticity field from continuous functions instead of a point-to-point estimation. Furthermore, the proposed measure of turbulence is integrative, accounting for dependencies across all conditions in the estimation process, thereby extending beyond traditional power spectral density methods. The model is depicted in Fig. 2.

We also anticipate that the latent temporal dynamics under study, represented by the kernel eigenfunctions, operate within a regime of near-criticality — a dynamical state poised near a phase transition, supporting flexible transitions between arousal states and heightened sensitivity to internal or external perturbations (Calderon et al. 2016). In support of this hypothesis, we interpret the observed multi-scale temporal structure as indicative of intrinsic neural complexity, consistent with systems near a continuous phase transition (Proekt et al. 2012). In such regimes, scale invariance (the absence of a characteristic time scale) allows fluctuations to span multiple temporal and spatial scales. Proekt et al. (2012) associate scale-invariant temporal dynamics with spontaneous behavior, a framework particularly relevant to our study, given the affective and internally modulated nature of the task. By interpreting turbulence as a proxy for this multi-scale complexity, our approach offers a principled way to characterize dynamical brain states beyond stimulus-locked responses, capturing the fluid, spontaneous structure of emotional-motor integration.

3 | MATERIALS AND METHODS

3.1 | Participants

We recruited 31 healthy volunteers (mean age, 27.6451 ± 6.5347 years; 21 female) based on the inclusion criteria of being right-handed, having normal or corrected-to-normal vision, normal hearing, and no history of neurological or psychiatric disorders. Participants reported a median of 10 years of musical education (range 1-10 years) despite not being music professionals or trained singers, fulfilling the required musical skills to perform the task. Musical scores were sent to the volunteers 48h prior to the experiment. All of them were requested to restrict caffeine

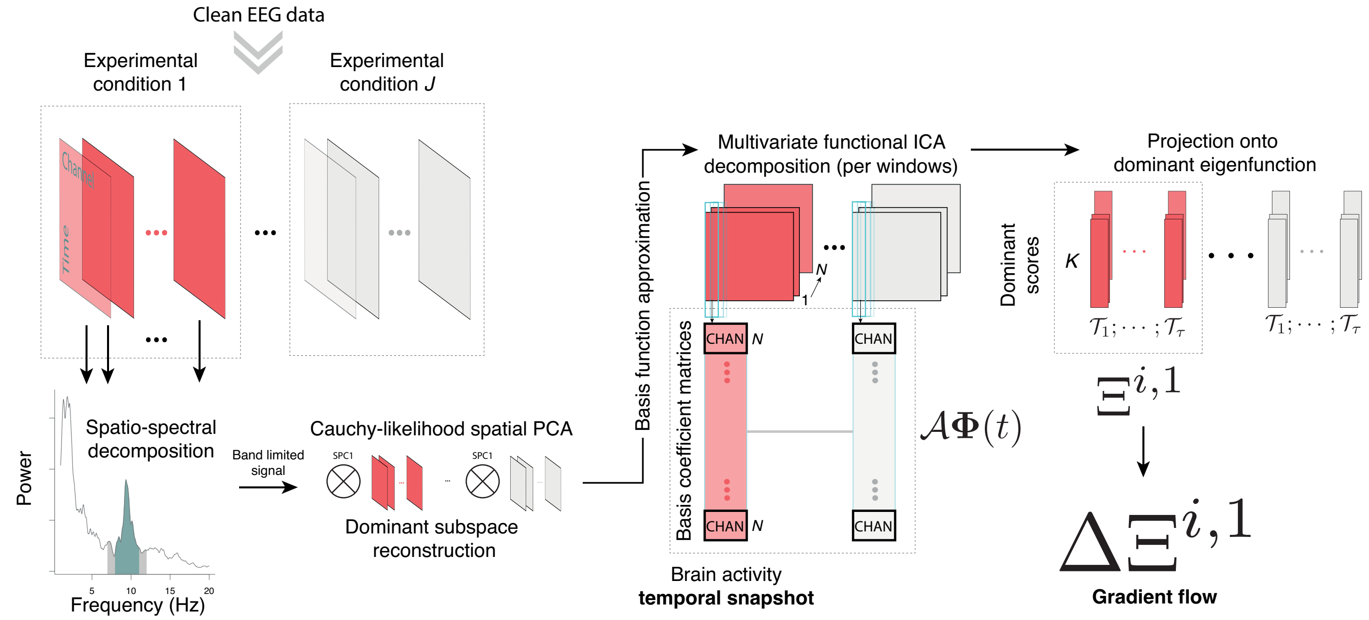

FIGURE 2 Spatiotemporal functional data model of EEG dynamics based on Pfaff (2009) generalized arousal (dominant dynamics), used to characterize turbulence-like patterns in brain activity.

intake or other stimulants on the data collection day. The full conduction of the experiment took ~2h, and a compensation of 20€ voucher was given upon completion. After screening, data from one participant was discarded due to bad electrode conductivity leading to a sample of $N = 30$ (mean age, 27.9333 ± 6.443 years; 20 female). This study was approved by the Ethics Committee of the Faculty of Arts and Philosophy of Ghent University (protocol no. 2022-33). Written informed consent was obtained from all subjects involved in the study.

3.2 | Screening

Before the experiment, all 31 initial recruits took part in a training and screening session with a laboratory technician and a musical expert. This was done to make sure participants were capable of singing the music correctly by heart and moving under a reasonable degree of freedom while using the equipment (mocap suit and VR headsets). After short testing of their ability to move, participants were asked to sing the song with the musical accompaniment under the guidance of the musical expert to check whether they were able to perform with fluency. Participants were naive to the purposes of the study, although they were informed that performance quality would not be under analysis, but rather their engagement and perceptions about the activity. Afterwards, instructions were read to the participants before starting the experiment.

3.3 | Task

Participants engaged in six tasks involving combinations of the following conditions: (1) singing vs. non-singing, (2) moving vs. non-moving, and (3) virtual agent (VA) interaction (expressive vs. robotic vs. mirroring vs. no VA). In moving tasks, participants were instructed to move freely to the music. In VA tasks, the VA was presented as conducting the music using either expressive gestures, robotic gestures, or mirroring the participants' movements in real time. The experiment was divided into two blocks, respectively, without and with VA. The first block included randomized two tasks: non-movement singing (NM.S.NA) and movement singing (M.S.NA). Prior to the second block, participants had a training familiarization with the VA, both conducting and mirroring their movements. Here, there were four randomized tasks: movement non-singing mirroring VA (M.NS.Mir) and movement singing mirroring VA (M.S.Mir), non-movement singing robotic VA (NM.S.Rob), and non-movement singing expressive VA (NM.S.Exp). At the beginning of each trial, instructions such as "Move. Sing along." or "Do not move. Do not sing." appeared in the virtual environment at 3.7 m distance from the participant's virtual view and 14 s before starting singing. A cue of 4 beats was included to signal the start of the music performance. Participants had to sing pronouncing "la" (instead of "ta") in order to limit artifactual effects in the EEG signal. The VR headset was worn throughout all tasks, including those without VA, to maintain consistency in sensory input and control for any potential confounding effects related to wearing the headset itself.

After performing each trial, participants were asked to rate their perceived level of emotional engagement, absorption (degree of immersiveness), control, and interaction (only for the second block) on a Likert

scale from 1 “low level” to 5 “high level”. They also rated their levels of stress at the beginning and the end of each block, with the intention of discarding trials in which levels above 3 were reported. No participant reported levels of stress > 3. The distribution of the ratings was: level 1 - 60.4838%, level 2 - 25.8064%, level 3 - 13.7096%. As in Hofmann et al. (2020), we further asked participants to indicate us if they felt general discomfort, nausea, dizziness, headache, blurred vision, and difficulty concentrating. Some participants felt discomfort in the nasal area due to the headset’s weight, which was cautioned by accommodating a cushion between the nose and the headset’s support zone. At the end of the experiment, participants were asked “Which of the agents (robotic, expressive, mirror, or none of them) do you believe had the most positive impact on your singing performance?” to reiterate their VA preferences, and to rate their involuntary urge to move during the conditions they were not allowed to.

3.4 | Data acquisition

Participants’ movement was recorded with a 16-infrared camera optical motion capture system (Qualisys, Sweden) using a sampling rate of 120 Hz. The acquisition software was the Qualisys Track Manager (QTM) 2023. Participants wore a suit where 42 reflective markers were placed following the Qualisys full body biomechanical model. This model was adopted due to its capability of producing realistic projections of the subjects’ movements.

EEG data was recorded at 1 kHz with ANT-Neuro *eeg*™ *mylab* systems using a 64 channel headset (10-10 system, with Ag/AgCl electrodes). One electrooculogram (EOG) electrode was placed below and next to the right eye. Recordings were conducted using a referential montage, with electrode CPz as reference. To reduce tension on electrode cables and allow unrestricted movements, the amplifier was positioned on an elevated table behind 1 m distance of the participant. Mobility was measured by freely swaying (with sufficient cable length) one step ahead and laterally. Impedance levels were monitored using the eego™ software to ensure they remained below 20 kΩ.

The VR headset was carefully placed over the EEG cap. Participants were equipped with HTC Vive Pro 2 headsets and followed the standard calibration procedure recommended by the manufacturer. The mocap data were streamed to a standard digital audio workstation software (Ableton Live 9) for synchronization with Unity (Unity Technologies, consumer version 2023.2.13) allowing the VA to mirror the participant’s movements. Vocal performance was recorded using a Shure Beta 87A microphone placed in the ceiling above the participant. Additionally, a decibel meter (UNI-T UT352) was employed to oversee and assess the volume levels before the commencement of the experiment, aiming to mitigate the impact of loudness.

3.5 | Stimuli

The virtual environment was designed in Unity. We used a gender-neutral VA in a room with plane size limited to 5$\times$10 m with low visual impact colors (Roy et al. 2021). Participants were standing in the middle of the room 3.7 m distance from the VA. Initially, a light gray cross was projected onto the middle of the scene to help participants fix their gaze on a point. The movements of the VA conducting robotically or expressively were recorded previously from a professional conductor instructed to perform the gesturing accordingly. In the robotic version, the conductor was restricted to a periodically repeated sequence of gestures denoting the tempo and time signature of the music, whereas in the expressive condition, the conductor embellished these basic temporal gestures with accompanying body sway to communicate expressive intentions, resulting in more expansive and varied movements (see accompanying video in the Supplementary Material). Auditory stimuli were the same as in our previous experiment (Vidal et al. 2024, see supplementary material online), where participants found it easy to memorize and adapt to their tonal range.

3.6 | Pre-processing of electrophysiological data

All pre-processing was performed in R (R Core Team 2021) using custom-made scripts. Routines were conducted separately for data recorded per participant and condition.

Detecting and removing artifacts in EEG signals during vocal tasks and body sway poses a complex challenge. Particularly, singing implies generation of artifacts from hypoglossal movement, involuntary clenching and by contractions of the neck and facial muscles. In addition, blink activity tends to be more prevalent in such conditions (Vidal et al. 2024). On the other hand, body movements can induce cable sway, muscle tension, and heightened heart rate variability. The critical mixture of artifactual sources over the sensor field rather requires that the method used for identification and removal suitably adjusts to their topological features in time and space. The approach considered here bears resemblance to a multi-band component analysis (Jonmohamadi and Muthukumaraswamy 2017), which allows targeting artifacts according to the bandwidth in which they arise more predominantly.

In a first stage, line noise interference was removed using a fourth-order Pei-Tseng notch filter centered at 50 Hz on the raw signal and FastICA (PCA whitening, parallel extraction with logcosh) was performed on the data high-pass filtered at 20 Hz (forward-backward 4th order Butterworth filter - 4Bw), a common low threshold for the spectral bandwidth of muscle activity (Muthukumaraswamy 2013). In order to select artifactual components, we inspected spatial topographies and applied wavelet shrinkage (MODWT-l4 (Percival and Mofjeld 1997)) to the vectors of the source matrix and visualized them using line references of the timings when the participants pronounced “la” to change

pitch. We enhanced their selection considering a median standard deviation threshold (as weighted by the number of channels containing absolute voltages > 100μV) of the norm of the transformed source vectors with the Teager-Kaiser operator (TKO) (Kaiser 1990). The selected components were individually backprojected for removal and denoised through wavelet shrinkage, as per the methodology outlined in Vidal and Aguilera (2025), aiming to minimize modulation and preserve brain activity to the greatest extent possible. On average, ~19 denoised and temporally-sparse high-frequency components were removed per subject, which is considered a reasonable number according to current investigations in the area (Muthukumaraswamy 2013, Liebisch et al. 2020).

Subsequently, adaptive notch (bandwidth: 0.1) filtering (Bedoyan et al. 2023) was performed on the data around spectral peaks exceeding the default threshold of 8 standard deviations using non-overlapping windows of a 50 Hz step from 48 Hz via FFT. We followed this protocol since muscle activity can mask sources of line noise, possibly produced by the VR headset system (Weber et al. 2021), while inducing their spectral distortion. Channels containing absolute voltages > 100μV above 20 Hz were denoised (MODWT-CI4) on the four coarsest decomposition levels (> 30 Hz) by shrinking to 0 the coefficients surpassing the universal threshold of their related TKO transformation. For the sake of smoothness, a Gaussian kernel was applied to these decomposition levels using Scott's bandwidth after performing the shrinkage. Pathological cases of noise-corrupted channels were visually inspected and reconstructed via spline spherical interpolation.

In a second stage, the data was referenced to robust average and FastICA was conducted on the broad-band pre-processed signal after a PCA reduction. A PCA usually enhances the estimation of high amplitude components corresponding to blinks, body movements or cable sway, by restricting their mixture with other PC's when ICA is performed. Outlying and sparsest spatial components were semi-automatically detected using the norms and the index of sparsity defined in Zima et al. (2012) on the vector columns of the estimated mixing matrix. To minimize the impact on brain activity, wavelet denoising (l4) was once again applied. The removal process was evaluated using depth statistics (Cuevas and Fraiman 2009) on the median absolute voltage and the norm of the Fourier spectrum (1-15 Hz) pooling all channels across subjects and conditions. Outlying channels and associated trials were inspected to further detect artifactual IC which were removed until sufficient depth consistency was achieved (only mastoids were left as residual outliers, which were not used in our analyses). Results were visually validated and, on average, ~1.7 artifactual components were removed from the signal. In a last round, the signal was again examined with ICA to identify residual artifactual activity and cardiogenetic components, whose detection is known to be improved under more stationary conditions.

A schematic overview of the full preprocessing pipeline is provided in Fig.3 to support clarity.

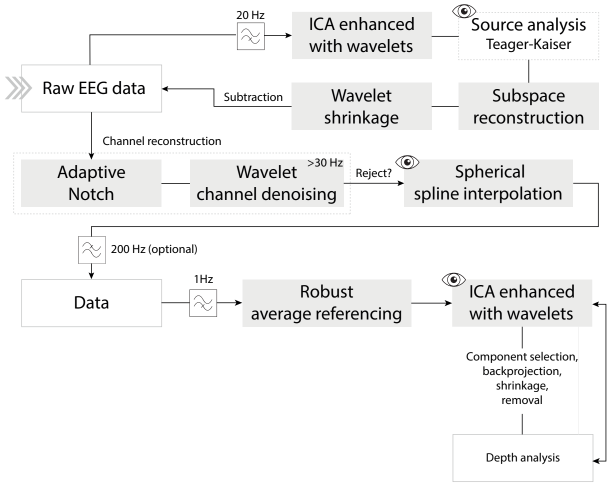

FIGURE 3 Schematic of the pre-processing pipeline. Visual inspection steps are marked with an eye icon.

3.7 | Robust subband estimation and EEG data representation

The pre-processed EEG data was filtered (4Bw) in the alpha (8-13 Hz) and high gamma (50-80 Hz) bands using spatio-spectral decomposition (SSD) (Nikulin et al. 2011). This technique aims to find linear filters maximizing power in the frequency band of interest while minimizing power in the neighboring "flanking" frequency bins. Prior studies have shown the ability of the technique to enhance the robustness and interpretability of results (Schaworonkow and Nikulin 2018, Hofmann et al. 2020, Cai et al. 2021), as well as to increase classification accuracy in BCI applications (Haufe et al. 2014). The SDD parameters were set as follows: bandpass signal $\pm$2 Hz, bandstop noise $\pm$1 Hz, bandpass noise $\pm$4 Hz. The number of SDD components (filters) was selected via the perpendicular line method, resulting in an average of ~36 and ~37.8 filters being retained for each band. Subsequently, Cauchy robust PCA (Fayomi et al. 2022) was applied to the filtered signal to represent the data in terms of the dominant spatial eigenvector. This technique, well-suited for high-dimensional data, utilizes a Cauchy likelihood instead of a Gaussian likelihood to ensure maximum robustness in component estimation.

3.8 | Hilbertian spatio-temporal model of dominant dynamics for turbulence analysis

To measure turbulence, we transformed EEG time series into functional components and analyzed their temporal fluctuations using a kurtosis-based decomposition. Let $X_{ij}(t_m, s_k)$ denote the EEG signal at time t_m, channel s_k, for participant i in condition j. The full dataset consists of N participants and J conditions recorded across K EEG channels and M time points. The matrices $(X_{ij})_{K\times M}$ were filtered using spatio-spectral

decomposition at the frequency band of interest and subsequently projected onto the first spatial component, extracted via PCA under a Cauchy likelihood model. Although the data is observed at $t_1, \ldots, t_M$ time points, we assume these are realizations of J spatio-temporal random variables on the space of square integrable functions $L^2_{T\times S}$. Given that our data comes in a wide format ($N \ll M$), the interval $T \subset \mathbb{R}$ representing the temporal domain is partitioned in $\mathcal{T}_1, \ldots, \mathcal{T}_\ell, \ldots, \mathcal{T}_\tau$ subintervals, possibly intersecting in a region of its domain.

Consider the basis expansions approximations at any $\mathcal{T}$ of $N{\cdot}K$ curves $X^j(t) = A^j\phi(t), t \in \mathcal{T}$ where A^j is $N \cdot K \times p$ coefficient matrix of the participant's curves pooled per channel and $\phi(t) = (\phi_1(t), \ldots, \phi_p(t))^\top$ is a basis of p functions. Note A^j represents sequences of matrices in the direction of the experimental conditions and X^j their respective vector of functions. Then, we concatenate all condition-specific curves into a single matrix. Thus, we will work with the expansion $\boldsymbol{X}(t) = \mathcal{A}\boldsymbol{\Phi}(t)$ where $\boldsymbol{X}(t) = [X^1(t); \ldots; X^J(t)]_{N\cdot K\times J}$, $\mathcal{A} = [A^1; \ldots; A^J]_{N\cdot K\times p\cdot J}$ and $\boldsymbol{\Phi}(t)$ is a matrix of size $p \cdot J \times J$ with J p-dimensional basis functions in its diagonal.

Following the standard ICA procedure, we map $\boldsymbol{X}(t)$ to orthogonality, that is,

$$\boldsymbol{X}(t) \to \mathbb{X}(t) : \ \mathrm{cov}(\mathbb{X}(t)) = \mathrm{id.},$$

using the factorization of the Gram matrix $\mathcal{G} = \int_\mathcal{T} \boldsymbol{\Phi}(t)\boldsymbol{\Phi}(t)^\top \mathrm{d}t$ (inner products between basis functions) and its inverse (see Vidal and Aguilera (2023)). Then, we consider the projections

$$\xi^j = \int_\mathcal{T} X^j(t)\psi^j_1(t)\mathrm{d}t, \tag{1}$$

where $\psi^j_1(t)$ is a function embedded in the dominant eigenfunction $\psi_1(t)$ obtained via spectral decomposition of the kurtosis kernel function admitting the basis expansion

$$\mathrm{kurt}(\mathbb{X})(t,\cdot) = \boldsymbol{\Phi}(\cdot)^\top \mathcal{G}^{-1/2} \underbrace{\left(\frac{1}{N\cdot K}\mathcal{G}^{1/2}\tilde{\mathcal{A}}^\top D\tilde{\mathcal{A}}\mathcal{G}^{1/2}\right)}_{\Sigma_{\tilde{\mathcal{A}}\mathcal{G}^{1/2}}} \mathcal{G}^{-1/2}\boldsymbol{\Phi}(t),$$

where $D = \mathrm{diag}\left(\tilde{\mathcal{A}}\mathcal{G}\tilde{\mathcal{A}}^\top\right)$, $\tilde{\mathcal{A}}$ is a coefficient matrix $\mathcal{A}$ after whitening and $\Sigma_{\tilde{\mathcal{A}}\mathcal{G}^{1/2}}$ is its kurtosis matrix. Note that the kurtosis kernel $\mathrm{kurt}(\mathbb{X})(t,\cdot)$ quantifies fourth-moment fluctuations over time.

We estimate $\psi^j_1(t)$ by solving the spectral decomposition of the empirical kurtosis matrix derived from the whitened coefficients. By solving the eigenvalue problem $\boldsymbol{\Phi}(t)^\top\Sigma_{\tilde{\mathcal{A}}\mathcal{G}^{1/2}}\mathcal{G}\boldsymbol{b}_s^\top = \kappa_s\boldsymbol{\Phi}(t)^\top\boldsymbol{b}_s^\top$, we get a set of eigenvalues $\kappa_1 \geqslant \cdots \geqslant \kappa_{p\cdot J}$ and associated eigenvectors $\boldsymbol{b}_s$ which allow to compose $\psi_s(t) = \boldsymbol{\Phi}(t)^\top\mathcal{G}^{-1/2}\boldsymbol{b}_s^\top$, the eigenfunctions of $\mathrm{kurt}(\mathbb{X})$ which have unit norm and are pairwise orthogonal. If we take the following division of the coefficients

$$[b_1, \ldots, b_p; b_{p+1}, \ldots, b_{2\cdot p}; \ldots; b_{(N\cdot K-1)\cdot p+1} \ldots, b_{N\cdot K\cdot p}],$$

one can easily obtain the dominant functions $\psi^j_1(t)$ by expanding each coefficient set by $\phi(t)$ and obtain (1).

In our model, the functions $\psi^j_1(t)$ are projected onto each univariate functional dataset X^j, thus preserving the participant's dimension across conditions, albeit at the cost of having non-uncorrelated projection scores. By subsequently performing at each $\mathcal{T}_\ell$ the above operations, we will get τ realizations of a discrete spatio-temporal random field, i.e., $\Xi^{ij} := \{\xi^{ij}_1; \ldots; \xi^{ij}_\ell; \ldots; \xi^{ij}_\tau\}$ where ξ^{ij}_ℓ is a univariate vector of K entries that has been reorganized participant-wise. The differentiation of Ξ^{ij} in time gives the gradient flow or fluctuation matrix and for all $\ell > 1$, the turbulence intensity is then defined as

$$\left\langle \|\xi^{ij}_\ell - \xi^{ij}_{\ell+1}\|^2 \right\rangle_\ell,$$

where $\langle\cdot\rangle_\ell$ indicates the average in the temporal direction. Therefore, turbulence intensity is the average squared difference between adjacent projection vectors across time. This yields a temporally resolved scalar measure of turbulence, reflecting the rate of change in dominant electrophysiological activity patterns over space.

3.9 | Turbulence model setup

For a system of overlapping windows, we determined a hop size of 20 ms according to the latencies that characterize interneuronal information transmission (Itoh et al. 2022). Window sizes of 500 and 100 ms for the alpha and gamma bands were respectively used to perform multivariate functional ICA with ZCA whitening (Vidal and Aguilera 2023). Note the spectral resolutions resolve at 2 Hz and 10 Hz, respectively, which allows sufficient distinction of oscillations within the narrow alpha band and adequate temporal precision for capturing the faster dynamics of the gamma band. Thus, this choice aimed to uphold a consistent ratio of neural oscillations in each window while, at the same time, mitigating the risk of numerical instabilities in the estimation of the covariance function in the functional ICA model. No improper ICA solutions were encountered, making it unnecessary to apply spectral truncation. We regressed out the data using B-spline basis functions keeping towards 0 the RMSE in the approximation. For the reconstruction of $\psi^j_1(\cdot)$ across all domain, an overlapping Gaussian window with a width factor of 4 was used, and the hop size was determined as half the window size.

3.10 | Pre-processing of motion capture data

Motion capture data was initially pre-processed in QTM 2023 (Qualisys AB, Sweden) for marker labeling, gap-filling, and trajectory smoothing (10 Hz low pass Butterworth filter). Marker trajectories were exported and movement velocity was then calculated as the first-order time-derivatives of the marker positions. Velocity data was then normalized across the three axes to produce the magnitude velocity of each marker. We then applied the Minimum Covariance Determinant estimator (Rousseeuw and van Driessen 1999) to the pre-processed spatial data, obtaining location estimates for each marker. These estimates were then median-averaged to calculate the movement velocity rate per participant and condition. Data from two participants were excluded from the MEM analyses due to technical issues with tracking.

3.11 | Post-hoc performance quality assessment

Following the same procedure as in Vidal et al. (2024), two musical experts performed an a posteriori quality assessment of the singing recordings. Audio recordings were presented in randomized order and evaluators rated them on a scale from 1 (very inaccurate) to 10 (very accurate) in the following items: intonation, rhythm, fluency, and memory. The discrepancy between the two evaluators on the singers' performance was not significant ($T^2 = 2.875, p = 0.095$). Following Koo and Li's reliability levels (Koo and Li 2016), we found good reliability of absolute agreement (Intraclass Correlation Coefficient = 0.864) and consistency (ICC = 0.865). No differences were found between the singing performance comparing the two experimental blocks ($T^2 = 1.0896, p = 0.3544$). Therefore, the evaluators reached the consensus that all participants were able to keep good performance levels.

3.12 | Statistics

Unless otherwise stated, statistical comparisons were performed using two-sided Wilcoxon tests for multiple pairwise comparisons between conditions, with Bonferroni-Holm correction: *$p < 0.05$, **$p < 0.01$, ***$p < 0.001$, ****$p < 0.0001$; n.s., not significant. We use W to denote the test statistic, p_{adj} for the adjusted p-value, and r for the effect size. Additional statistical procedures are described directly in the corresponding sections.

4 | RESULTS

4.1 | Self-reports

Graphical representation of the self-report results is shown in Fig. 4. Emotional engagement was rated higher in the mirroring (M.NS.Mir, $W = 668$, $p_{adj} = 0.01$, $r = 0.436$; and M.S.Mir, $W = 187$, $p_{adj} = 0.0005$, $r = 0.536$) and expressive (NM.S.Exp, $W = 242$, $p_{adj} = 0.01$, $r = 0.408$) conditions compared to the robotic condition (NM.S.Rob). Similar results were found for mirroring (M.S.Mir, $W = 214$, $p_{adj} = 0.005$, $r = 0.465$; and M.NS.Mir, $W = 242$, $p_{adj} = 0.018$, $r = 0.408$) and expressive conditions (NM.S.Exp, $W = 254$, $p_{adj} = 0.027$, $r = 0.389$) compared to the non-movement singing no-avatar condition (NM.S.NA). Absorption was rated higher in the singing mirror (M.S.Mir, $W = 224$, $p_{adj} = 0.006$, $r = 0.456$) and expressive conditions (NM.S.Exp, $W = 241$, $p_{adj} = 0.012$, $r = 0.427$) compared to the robotic condition (NM.S.Rob). Absorption was also rated higher in the mirroring (M.S.Mir, $W = 197$, $p_{adj} = 0.002$, $r = 0.500$; and M.NS.Mir, $W = 247$, $p_{adj} = 0.022$, $r = 0.400$) and expressive conditions (NM.S.Exp, $W = 209$, $p_{adj} = 0.003$, $r = 0.478$) compared to NM.S.NA. In both variables, no significant effects were found for the movement singing no-agent condition (M.S.NA). For the variable control, no significant effects were found. Interaction levels with the virtual avatar were rated higher for the mirroring (M.S.Mir, $W = 110$, $p_{adj} < 0.0001$, $r = 0.678$; and M.NS.Mir, $W = 764$, $p_{adj} < 0.0001$, $r = 0.639$) and expressive conditions (NM.S.Exp, $W = 119, p_{adj} < 0.0001$, $r = 0.668$) compared to the robotic condition (NM.S.Rob). Furthermore, in the qualitative comments, 21 participants reported that the expressive VA had a positive impact on their performance, while 9 preferred the VA mirroring their movements.

In summary, the self-report results indicate that emotional engagement, absorption, and VA interaction were consistently higher in conditions presenting natural human movement (expressive and mirroring) compared to conditions presenting robotic movement or no avatar at all.

4.2 | Dominant brain dynamics during motor-related emotional arousal states exhibit multiscale fluctuation patterns consistent with turbulence

Turbulent systems can exhibit multiscale organization and heavy-tailed dynamics, which approximate scale invariance over certain ranges, though they deviate from the strict scaling laws seen in critical phenomena (Deco et al. 2025). This behavior reflects a balance between order and disorder in out-of-equilibrium dynamics, supporting flexible transitions across spatial and temporal scales. In the brain, such multiscale temporal structure has been linked to critical-like dynamics and spontaneous behavior, particularly during emotionally or behaviorally engaged states (Proekt et al. 2012). Following Daffertshofer et al. (2018), we divided the reconstructed dynamical modes $\psi_1^j(t)$ into multiscale non-overlapping segments and modeled the distributions of temporal fluctuations using log-normal fits, which provide an effective approximation of scale-invariant behavior, especially in the presence of heavy-tailed dynamics (Buzsáki and Mizuseki 2014). A total of 54 levels, factors of 56 s (the duration of the musical piece) ranging from 28 to 0.002 s were used in the analyses. We then calculated the mean fluctuation for each segment (see formula no. 4 in Daffertshofer et al. (2018)) and averaged them at each level. To study the presence of scale invariance, we opted for fitting a continuous log-normal distribution instead of a power law. This choice was motivated by the fundamental nature of lognormal behavior in reflecting the complex structural and functional organization of the brain (Buzsáki and Mizuseki 2014). Additionally, log-normal distributions offer improved modeling of tail behavior, particularly in the presence of extreme values. Results suggest that dominant brain dynamics during motor-related emotional arousal states are (in general) scale invariant (Tab. 1). Levels of significance were derived following goodness of fit (GOF) tests based on the Kolmogorov-Smirnov statistic (Clauset et al. 2009), via bootstrapping (1000 iterations) on the data. We observe that the results for the gamma band are statistically more consistent, exhibiting an overall better GOF and showing less sensitivity to the level of significance. By showing that the dominant temporal components $\psi_1^j(t)$

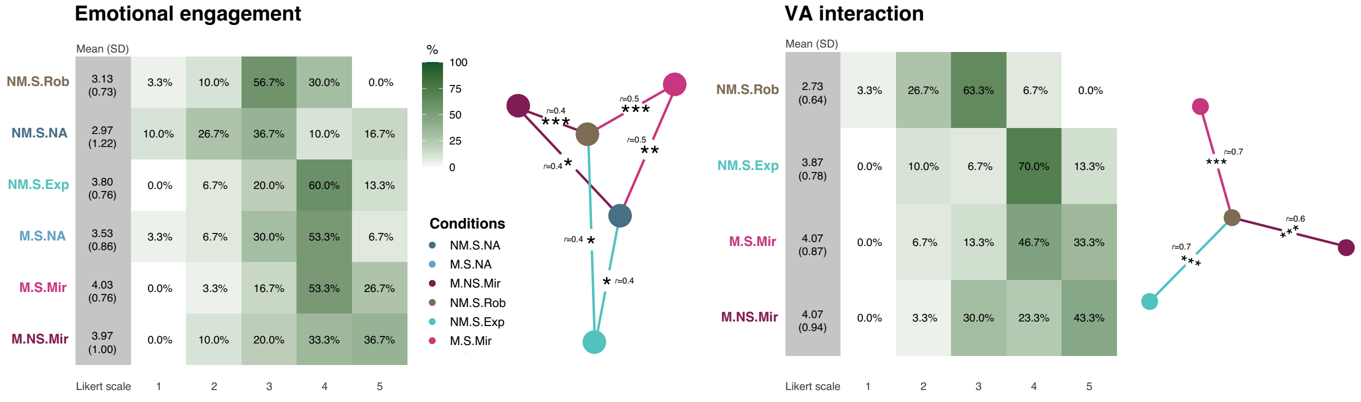

FIGURE 4 Behavioral data: Percentage distributions of the participants' emotional engagement and VA interaction ratings (1 to 5) across task conditions with accompanying graphs of the rated items directed with colors towards the condition with higher mean rates with the indication of the test significance and effect sizes.

TABLE 1 The table shows the results of fitting a continuous log-normal distribution to the temporal fluctuation structure of the reconstructed components. The parameters μ and σ represent the estimated mean and standard deviation of the fitted distribution. The *p*-values and goodness-of-fit (GOF) statistics are derived from a bootstrapped Kolmogorov–Smirnov test (1000 iterations), evaluating the null hypothesis that the empirical distribution is consistent with a log-normal model. A non-significant p-value (p > 0.05) indicates that log-normality cannot be ruled out.

Condition (alpha band)	*p*-value	GOF	μ	σ
NM.S.NA	0.0060	0.1254	2.3412	0.0181
M.S.NA	0.0960	0.1150	2.3479	0.0061
M.NS.Mir	0.1320	0.0922	2.2919	0.0294
NM.S.Rob	0.1290	0.0960	2.3145	0.0198
NM.S.Exp	0.0660	0.0967	2.2988	0.0278
M.S.Mir	0.8140	0.0868	2.3333	0.0017
**Condition (gamma band)**	*p*-value	**GOF**	μ	σ
NM.S.NA	0.7350	0.1090	2.5173	0.0010
M.S.NA	0.1910	0.1165	2.5505	0.0026
M.NS.Mir	0.6000	0.1072	2.5442	0.0015
NM.S.Rob	0.2850	0.1098	2.5669	0.0024
NM.S.Exp	0.3370	0.1110	2.5660	0.0021
M.S.Mir	0.7280	0.0916	2.5532	0.0017

exhibit statistical self-similarity across scales, we provide evidence that the underlying brain dynamics share key features of turbulent regimes.

4.3 | Dominant brain dynamics during motor-related emotional arousal states are distinctly turbulent

As a kurtosis value of 3 corresponds to that of a Gaussian distribution, this specific threshold can be taken to determine a cutoff point to discern the transition from the stability inherent in a Gaussian scenario to a turbulent state of non-Gaussian behavior. To investigate the presence of turbulence, our approach involves an information measure based on the differential entropy called Entropic Normalized Information Distance (ENID) (Bruni et al. 2020), that aims to separate realizations of a random variable into two (as much as possible) statistically independent subsets: here, those kurtosis coefficients of ξ_ℓ^{ij}'s > 3 (for all i, j) attracted to the vicinity of 3, and those who depart from it. By applying ENID to the inverse of the coefficients (the result should then reflect the separation point after 3), divergence from a Gaussian setting occurs at a kurtosis coefficient threshold of 8.2453 and 6.6926 for the alpha and gamma bands respectively. This indicates that turbulence is more prominent in the gamma band, as evidenced by ENID separating faster from 3. We added a baseline condition (no movement, no singing, just listening to the music) to validate our results against a commonly controlled EEG task in music research. As our model yields the maximized kurtosis in time-space for each condition rather than each participant, the counts of values exceeding this threshold are reported on a per-condition basis (proportion of counts alpha; gamma): Baseline 57.677; 42.0606%, NM.S.NA 80.071; 59.1534%, M.S.NA 31.3266; 55.9082%, M.NS.Mir 38.3972; 62.1736%, NM.S.Rob 60.2062; 37.164%, NM.S.Exp 62.2641; 38.2332%, M.S.Mir 31.7516; 56.4618%. In summary, high gamma-band turbulence increased in conditions involving motor execution and/or emotional engagement (e.g., NM.S.NA, M.S.NA, M.NS.Mir, M.S.Mir). In contrast, alpha-band turbulence was highest in conditions with lower motor demands (e.g., NM.S.NA, NM.S.Exp, NM.S.Rob).

4.4 | Lower turbulence intensity reflects higher level of motor-related emotionality

We initially compared potential differences in turbulence intensity within the alpha and gamma bands (results are reported on Tab. 2 and Fig. 5). Our analysis across both bands revealed varying significance levels, particularly noteworthy in the cases of NM.S.NA and NM.S.Rob, which exhibited elevated contributions to turbulence intensity with respect to the rest of the conditions at the baseline level.

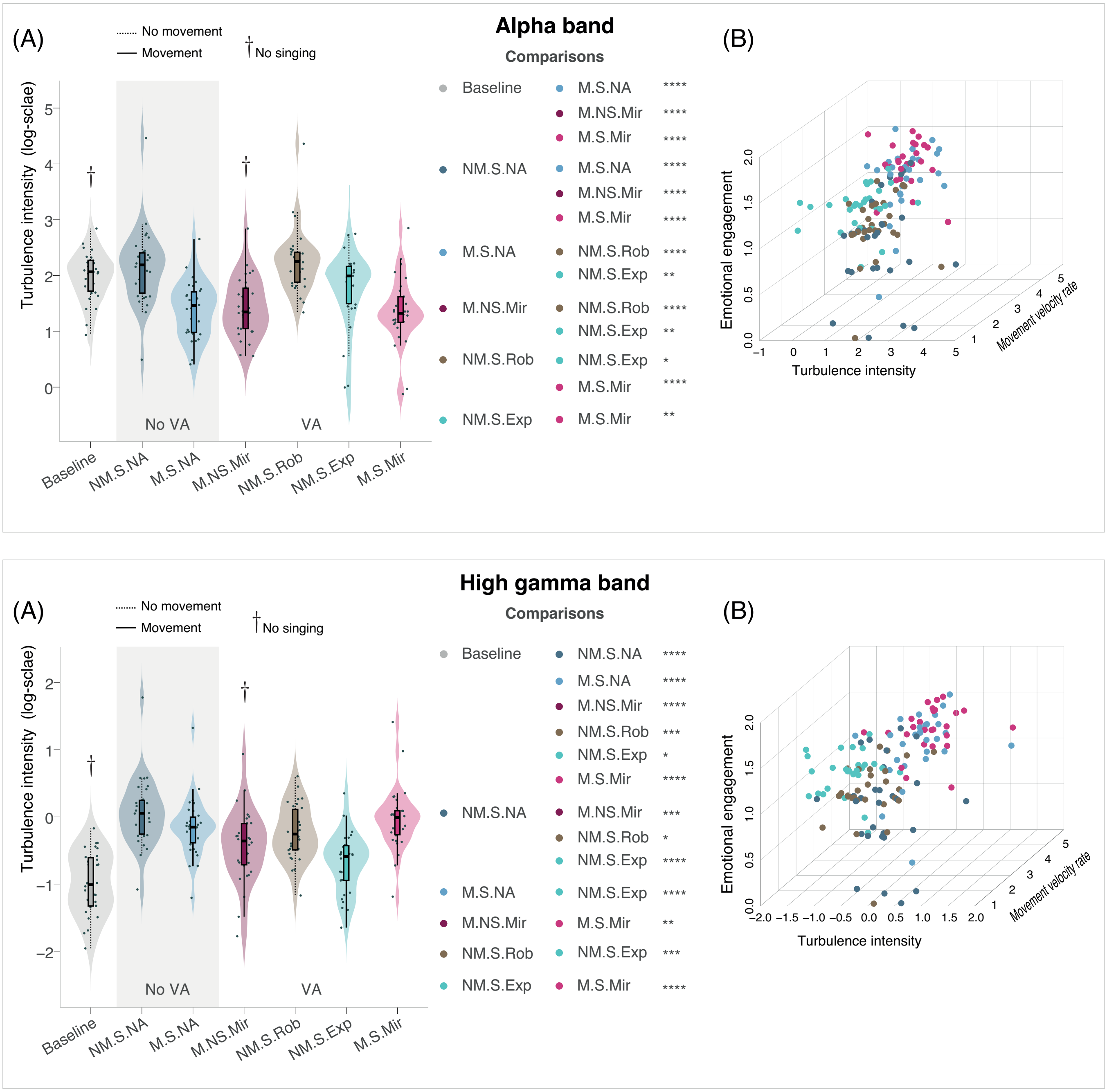

FIGURE 5 Turbulence analysis of EEG signal. *(A)* Boxplots of turbulence intensity across conditions in alpha (8-13 Hz) and high gamma (50-80 Hz). The centre line of each boxplot represents the data median and the bounds of the box show the interquartile range. The whiskers represent the bottom 25% and top 25% of the data range. The baseline condition is NM.NS.NA, only listening to the music. *(B)* 3D scatterplots showing interactions between the variable emotional engagement, turbulence intensity and movement velocity rates in a log-scale.

Further, turbulence intensity was higher for the mirroring conditions (M.NS.Mir, M.S.Mir) and M.S.NA in the gamma band, while NM.S.Exp exhibited the lowest level. For the current results, empirical power of Wilcoxon tests was assessed using simulations on non-Gaussian data $\exp(1) - 1$ to validate the sensitivity of the effects (in Tab.2, if $N < 30$ it means 100% of reliability) which proved to be highly reasonable. To confirm that the reported effects are not isolated and support the structure of the comparisons shown, we ran Friedman tests as non-parametric omnibus tests which yielded highly significant results (alpha band, $p < 0.00001, \chi^2 = 115.93$, Kendall's $W = 0.68$; gamma band $p < 0.00001, \chi^2 = 120.6$, Kendall's $W = 0.7$).

Having established overall turbulence intensity differences across conditions, we next examined whether these differences could be explained by individual-level behavioral measures, including emotional

TABLE 2 Mean turbulence velocity levels on the alpha and high gamma band. Statistical comparisons.

**Alpha band**	W	p_{adj}	r	Power	N
Baseline - M.S.NA	743	<0.0001	0.5592	0.9610	20
Baseline - M.NS.Mir	728	<0.0001	0.5306	0.9300	20
Baseline - M.S.Mir	742	<0.0001	0.5573	0.9470	20
NM.S.NA - M.S.NA	757	<0.0001	0.5859	0.9730	20
NM.S.NA - M.NS.Mir	744	<0.0001	0.5611	0.9390	20
NM.S.NA - M.S.Mir	765	<0.0001	0.6012	0.9670	20
M.S.NA - NM.S.Rob	94	<0.0001	0.6794	0.9020	10
M.S.NA - NM.S.Exp	222	0.0010	0.4351	0.7490	30
M.NS.Mir - NM.S.Rob	108	<0.0001	0.6527	0.8700	10
M.NS.Mir - NM.S.Exp	240	0.0030	0.4008	0.6910	30
NM.S.Rob - NM.S.Exp	606	0.0330	0.2977	0.6510	30
NM.S.Rob - M.S.Mir	803	<0.0001	0.6737	0.8670	10
NM.S.Exp - M.S.Mir	676	0.0010	0.4313	0.7660	30
**High gamma band**					
Baseline - NM.S.NA	37	<0.0001	0.7882	0.9960	10
Baseline - M.S.NA	77	<0.0001	0.7119	0.9550	10
Baseline - M.NS.Mir	189	0.0002	0.4981	0.9030	20
Baseline - NM.S.Rob	108	<0.0001	0.6527	0.9310	10
Baseline - NM.S.Exp	289	0.0270	0.3072	0.6820	30
Baseline - M.S.Mir	48	<0.0001	0.7672	0.9890	10
NM.S.NA - M.NS.Mir	698	0.0003	0.4733	0.9320	30
NM.S.NA - NM.S.Rob	616	0.0240	0.3168	0.6650	30
NM.S.NA - NM.S.Exp	807	<0.0001	0.6813	0.8790	10
M.S.NA - M.NS.Mir	600	0.0390	0.2862	0.5850	30
M.S.NA - NM.S.Exp	750	<0.0001	0.5725	0.9590	20
M.NS.Mir - M.S.Mir	231	0.0020	0.4179	0.8400	30
NM.S.Rob - NM.S.Exp	704	0.0003	0.4847	0.8920	20
NM.S.Exp - M.S.Mir	116	<0.0001	0.6374	0.8180	10

engagement and movement velocity rates. We used a mixed-effects model (MEM) via restricted maximum likelihood leveraging subject/-condition variation, focusing exclusively on the singing conditions (only Baseline and M.NS.Mir were excluded). The response variable was turbulence intensity, while the predictors included the rated level of emotional engagement (EE) and the movement velocity rate (MVR) (see Materials and Methods), all in a log-scale. For the alpha band, the model output (AIC = 239.5063, Log-Likelihood/LL = -114.7531) indicates a significant effect of EE ($\beta = -0.0487, p = 0.048$) and strong effect of MVR ($\beta = -0.2194, p < 0.0001$) on turbulence intensity with intercept correlation (IC) of -0.728 for EE and -0.219 for MVR. Note that IC measures the degree of association between the intercept and each fixed effect, which is often used as a measure for the effect size. The conditional $R_c^2 = 0.6399$, reflecting the total variance explained by both fixed effects and random intercepts, indicates that the model accounts for a substantial and consistent portion of the variance. No random slopes were included in our models to avoid complexity and overfitting. In the gamma band, the model output (AIC = 183.3267, LL = -86.6633, $R_c^2 = 0.5395$) indicates a significant effect of EE ($\beta = -0.3622, p = 0.0063$) and MVR ($\beta = 0.0987, p = 0.0001$) with a IC of $-0.736, -0.224$ respectively.

Therefore, turbulence intensity varied across conditions and was modulated by EE (negatively in both bands) and MVR (negatively in alpha, positively in gamma). Fig. 5B shows current interactions in 3D scatterplots. The significance levels stayed consistent when a genre predictor was included in the models.

4.5 | Lower turbulence intensity in higher gamma band corresponds to level of emotionality and emotional motor control

In the current MEM, we introduced a combined effect of (MVR + Task Demand), where Task Demand (TD) is a predictor that assigns values of 1, 2, or 3 to the conditions according to level of demand (moving, singing, interacting with the VA or any combination of them). For the alpha band, the model output (AIC = 230.794, LL = -110.3972, $R_c^2 = 0.6661$) indicates a strong effect of MVR + TD ($\beta = -0.2066, p < 0.000$) on turbulence intensity with an IC of -0.191. No significant effects were found for EM ($\beta = 0.0274, p = 0.1737$). For the gamma band, the model output (AIC = 188.8453, LL = -89.4226, $R_c^2 = 0.5124$) indicates significant effects of EE ($\beta = -0.3442, p = 0.0085$) and MVR + TD ($\beta = 0.0716, p = 0.001$) on turbulence intensity with an IC of -0.726 for EE and -0.202 for MVR + TD. Finally, we tested whether the observed effects in the high gamma range could be further explained by subjective emotional experience and its interaction with perceived control. By adding the interaction term EM:Control to the model (AIC = 191.5129, LL = -90.7564, $R_c^2 = 0.51$), we found effects of EE:Control

($\beta = -0.2143, p = 0.0204$) and MVR + TD ($\beta = 0.0617, p = 0.0014$) on turbulence intensity with an IC of -0.569 and -0.328, respectively.

In summary, turbulence intensity in the high gamma band was significantly predicted by the combined effects of task demand and movement velocity, as well as by the interaction between emotional engagement and perceived control. The significance levels stayed consistent when a genre predictor was included in the models.

5 | DISCUSSION

We have shown that, during motor-related emotional arousal, dominant EEG dynamics display signatures of turbulence consistent with a near-critical regime. Evidence presented here suggests that turbulence modulation in alpha band activity (8-13 Hz) is primarily driven by motor function and associated level of task demand. We found a dual behavior between alpha and high gamma band (50-80 Hz) dynamics that suggests that, even in the absence of overt bodily movement, emotionality is revealed by the integrated interpretation of both bands.

While alpha-band activity has been a longstanding subject of psychophysiological investigations, its systematic examination in relation to motor-specificity of alpha emotional modulation has only emerged in a few studies (Genzer et al. 2018, Siqi-Liu et al. 2018, Hofmann et al. 2020, Washburn et al. 2019, Wang et al. 2023). Extensive work suggests that alpha oscillations play a crucial role in optimizing cognitive resources by selectively dampening neural responses to non-pertinent information (Foxe and Snyder 2011). Decline of alpha power in the extended motor system has been shown to engage in neuronal spiking, whereas increased alpha power exhibits phase synchronization due to rhythmic inhibition of neuronal firing. This supports the idea that alpha oscillations serve as an informative reflection of the motor system's state, acting as predictive markers of the overall performance (Haegens et al. 2011, Halgren et al. 2019). Our findings suggest that alpha turbulence down-modulation, at least in terms of the dominant dynamics under study, is produced by the influences of motor functionality in relation to the level of task demand. This is clearly illustrated by the gradual decrease in turbulence intensity, progressing from no movement during singing, to singing while observing the VA conducting, and ultimately singing while mirroring the movement on the VA (Fig.5A). As previous investigations have shown (see Hofmann et al. (2020)), this down-modulation is specifically observable at the occipito-parietal electrodes (Fig.6). We speculate that this effect could possibly be linked to volume conduction arising from low-frequency cholinergic axonal diffusion during states of motor planning and movement (Reimer et al. 2016, Lohani et al. 2022, Vidal et al. 2024), or to recent evidence on the control of inhibitory neurons by the cholinergic system (Nair et al. 2023). The observed contrast of alpha turbulence intensity in the NM conditions (Fig.5A and Fig.6) is in agreement with previous findings (Genzer et al. 2018, Ross et al. 2021) suggesting that synchronization of mu activity (8–12 Hz) in left and midline somatomotor area indicates active inhibition of motor urges, which here aligns with participants' inclination to move during the various singing tasks (25 out of 30 participants reported levels ≥ 3 of involuntary urge to move in restricted movement conditions). The influence of motor afferents in alpha modulation is reinforced by the fact that motor brain regions, particularly the basal ganglia, cerebellum, and premotor cortices including area 55b (a restricted region in the right hemisphere), are consistently activated during music listening, even in the absence of overt bodily movement (Siman-Tov et al. 2022).

Although our study did not generate ample statistical evidence to validate turbulence alpha down-modulation as a robust signature of emotional arousal, we found that high gamma activity was a more suitable candidate for this purpose. Aside from the effects of movement and task demand, results in gamma turbulence modulation suggest balanced contributions of emotional engagement and emotional motor control. Particularly relevant to these findings is that high-frequency brain activity supports the existence of GA function (Calderon et al. 2016, Gao et al. 2019), often evidenced in humans as a surge of gamma power linked to conscious processing during comatose states (Xu et al. 2023). Based on our results (Fig.5A), we hypothesize that GA function plays a complex role in shaping motor-related emotional arousal, reflecting a mind-brain-body interplay as in Gordon et al. (2023) (i.e., coordinating and integrating motor functions to convey emotional expression). Remarkably, participants' sense of emotion was significantly correlated to down-modulation of turbulence in the gamma range, apparently implying a process of embodiment (Leman 2008), considering that this was observed in conditions with movement and/or watching natural movement (M.NS.Mir, NM.S.Exp). In complement, NM.S.Exp was the condition in which participants perceived the greatest increase in their performance level, reinforcing the connection between gamma turbulence down-modulation and emotional motor control. The observed contrast in condition M.S.Mir, where alpha turbulence decreases while gamma activity intensifies, suggests an increase in attentional demands (Kim et al. 2017, Stitt et al. 2018), possibly linked to heightened noradrenergic axonal activity (Dahl et al. 2022, Noei et al. 2022). Interestingly, this dual behavior appears to be compatible with participants' reported level of emotional engagement. Recent investigations have established connections between attention and emotion employing models that link noradrenergic and cholinergic activity to distinct pupillary signatures (Bang et al. 2023, Vidal et al. 2024).

Some limitations were identified in the current research. While current results in the high-gamma band are consistent and interpretable, one should not rule out the possibility that they are, to some extent, sensitive to the presence of residual muscle artifacts. Furthermore, identifying specific contributions of emotional arousal, at a brain level, becomes increasingly challenging considering the overlapping cognitive, motor, and emotional processes involved in the task under study. This is, however, an idiosyncrasy of naturalistic designs implementing close to reality activities and allowing for greater behavioral freedom of the participants. Due to the intermittent and chaotic nature of turbulence, tailored measures of dissipation, metastability, functional connectivity,

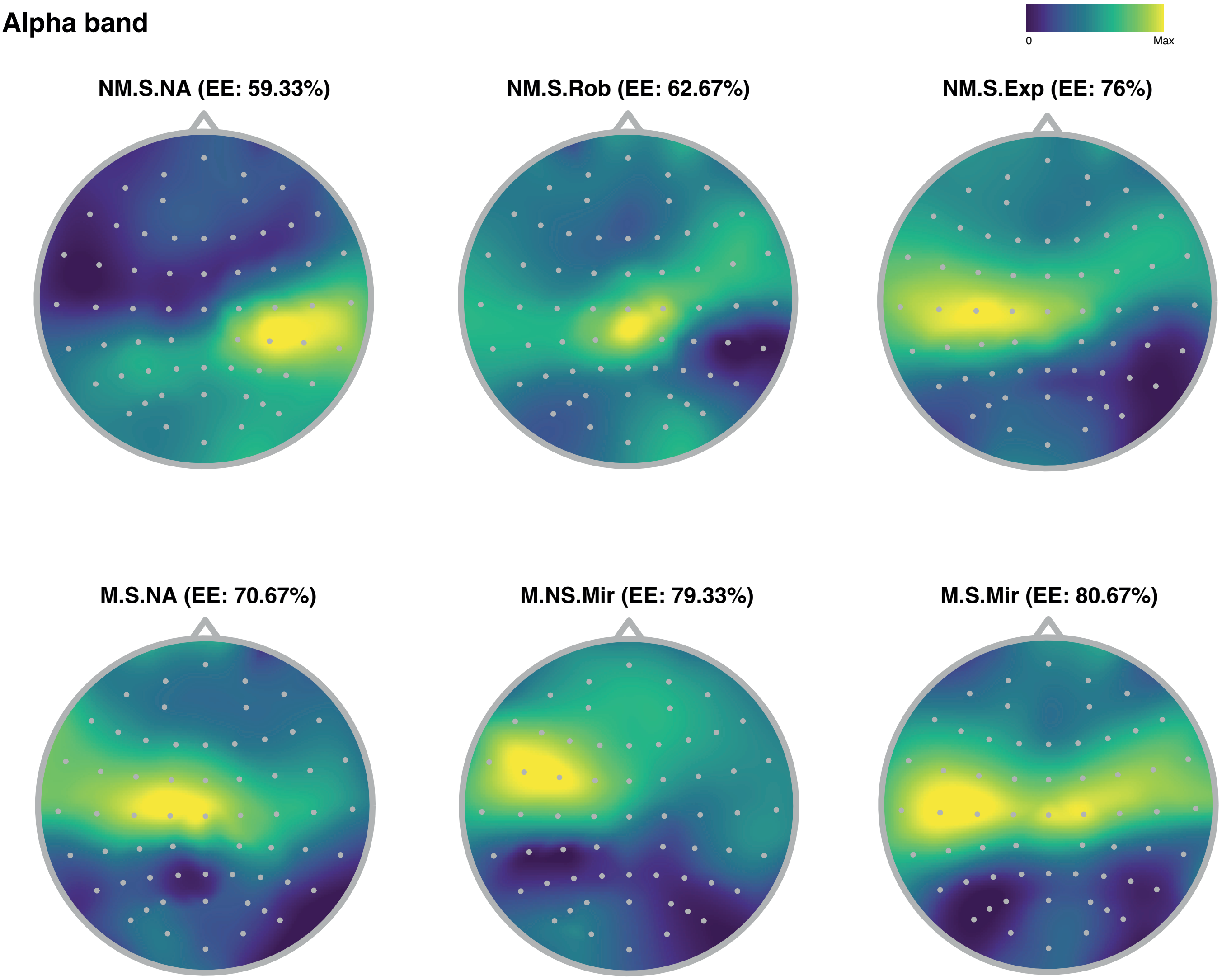

FIGURE 6 Spatial patterns in alpha band during the different motor conditions. Colours represent the absolute value of the participant-average of the turbulence scores $\langle \xi_\ell^{ij} \rangle_\ell$ after application of spherical spline surface Laplacian (Carvalhaes and de Barros 2015). This technique is used to mitigate artifactual volume conduction effects and localize interesting cortical areas involved in the cognitive task under examination. High turbulence levels are observed in the sensorimotor area, particularly in central and centro-left lateral regions, during conditions involving movement and/or the visualization of movement. The occipito-parietal region shows a reduction in turbulence as the perceived level of emotional engagement (EE) and physical task demand increases. Percentages represent the mean value of the EE ratings.

among others, will help find precise topographical descriptors of such emotional states. Secondly, considering that lower turbulence levels occurred in conditions involving just singing or just watching movement, hence implying lower cognitive workload, our study emphasizes the need for further clarification of the role of attention in emotional processing during motor tasks. Using immersive VR is necessary to enhance the trade-off between internal and external validity in psychophysiological research. Although current VR still constitutes a reductionist version of in-person musical interactions, our work unveils that it can evoke emotionality in simulation contexts when genuine ones are intangible, offering promising clinical and educational applications (Pozeg et al. 2017, Campo et al. 2023).

AUTHOR CONTRIBUTIONS

Conceptualization, M.V. and M.L.; methodology, M.V. and B.v.K.; software, M.V. and B.v.K.; validation, M.V. and A.A.; formal analysis, M.V., N.M., A.A. and M.L.; data curation, M.V.; writing—original draft preparation, M.V.; writing—review and editing, N.M., A.A., B.v.K., T.F. and M.L.; supervision, A.A., T.F. and M.L.; funding acquisition, A.A. and M. L.. All authors have read and agreed to the published version of the manuscript.

ACKNOWLEDGMENTS

We are grateful to T. Fujioka (Stanford University), D. Marinazzo (Ghent University), V. Nikulin (Max Planck Institute for Human Cognitive and Brain Sciences), for their suggestions on the data pre-processing and analysis, and to C. Gener her support during data collection. The authors also thank the referees for their constructive and helpful comments, which improved the original manuscript.

FINANCIAL DISCLOSURE

None reported.

CONFLICT OF INTEREST

The authors declare no potential conflict of interests.

REFERENCES

Aguilera, A.M., Ocaña, F.A. & Valderrama, M.J. (1997) An approximated principal component prediction model for continuous-time stochastic processes. *Applied Stochastic Models and Data Analysis*, 13, 61–72.
URL https://doi.org/10.1002/(SICI)1099-0747(199706)13:2<61::AID-ASM296>3.0.CO;2-I

Arroyo, S., Lesser, R.P., Gordon, B., Uematsu, S., Jackson, D. & Webber, R. (1993) Functional significance of the mu rhythm of human cortex: an electrophysiologic study with subdural electrodes. *Electroencephalography and Clinical Neurophysiology*, 87, 76–87.
URL https://doi.org/10.1016/0013-4694(93)90114-B

Ballinger, E.C., Ananth, M.R., Talmage, D.A. & Role, L.W. (2016) Basal forebrain cholinergic circuits and signaling in cognition and cognitive decline. *Neuron*, 91(6), 1199–1218.
URL https://doi.org/10.1016/j.neuron.2016.09.006

Bang, D., Luo, Y., Barbosa, L.S., Batten, S.R., Hadj-Amar, B., Twomey, T. et al. (2023) Noradrenaline tracks emotional modulation of attention in human amygdala. *Current Biology*, 33(22), 5003–5010.e6.
URL https://doi.org/10.1016/j.cub.2023.09.074

Bangert, M. & Altenmüller, E. (2003) Mapping perception to action in piano practice: a longitudinal dc-eeg study. *BMC Neuroscience*, 4, 26–26.
URL https://doi.org/10.1186/1471-2202-4-26

Bartoli, E., Kelly, S.M., Schofield, P.J., Pezaris, J.W. & Bosking, B. (2019) Functionally distinct gamma range activity revealed by stimulus tuning in human visual cortex. *Current Biology*, 29(21), 3562–3570.e5. doi:10.1016/j.cub.2019.08.034.
URL https://doi.org/10.1016/j.cub.2019.08.034

Bedoyan, E., Reddy, J.W., Kalmykov, A., Cohen-Karni, T. & Chamanzar, M. (2023) Adaptive frequency-domain filtering for neural signal preprocessing. *NeuroImage*, 284, 120429.
URL https://doi.org/10.1016/j.neuroimage.2023.120429

Berkooz, G., Holmes, P. & Lumley, J.L. (1993) The proper orthogonal decomposition in the analysis of turbulent flows. *Annual Review of Fluid Mechanics*, 25, 539–575.

Bowling, D.L., Gahr, J., Ancochea, P.G., Hoeschele, M., Canoine, V., Fusani, L. et al. (2022) Endogenous oxytocin, cortisol, and testosterone in response to group singing. *Hormones and Behavior*, 139, 105105–105105.
URL https://doi.org/10.1016/j.yhbeh.2021.105105

Bruni, V., Cioppa, L.D. & Vitulano, D. (2020) An automatic and parameter-free information-based method for sparse representation in wavelet bases. *Mathematics and Computers in Simulation*, 176, 73–95.
URL https://doi.org/10.1016/j.matcom.2019.09.016

Buzsáki, G. & Mizuseki, K. (2014) The log-dynamic brain: how skewed distributions affect network operations. *Nature Reviews Neuroscience*, 15, 264–278.
URL https://doi.org/10.1038/nrn3687

Cai, Z., Sohrabpour, A., Jiang, H., Ye, S., Joseph, B., Brinkmann, B.H. et al. (2021) Noninvasive high-frequency oscillations riding spikes delineates epileptogenic sources. *Proceedings of the National Academy of Sciences*, 118(17), e2011130118.
URL https://doi.org/10.1073/pnas.2011130118

Calderon, D.P., Kilinc, M., Maritan, A., Banavar, J.R. & Pfaff, D.W. (2016) Generalized cns arousal: An elementary force within the vertebrate nervous system. *Neuroscience Biobehavioural Reviews*, 68, 167–176.
URL https://doi.org/10.1016/j.neubiorev.2016.05.014

Campo, A., Michałko, A., Kerrebroeck, B.V., Stajic, B., Pokric, M. & Leman, M. (2023) The assessment of presence and performance in an ar environment for motor imitation learning: A case-study on violinists. *Computers in Human Behavior*, 146, 107810.
URL https://doi.org/10.1016/j.chb.2023.107810

Candia-Rivera, D., Catrambone, V., Thayer, J.F., Gentili, C. & Valenza, G. (2022) Cardiac sympathetic-vagal activity initiates a functional brain–body response to emotional arousal. *Proceedings of the National Academy of Sciences*, 119(21), e2119599119.
URL https://doi.org/10.1073/pnas.2119599119

Carvalhaes, C. & de Barros, J.A. (2015) The surface laplacian technique in eeg: Theory and methods. *International Journal of Psychophysiology*, 97(3), 174–188.
URL https://doi.org/10.1016/j.ijpsycho.2015.04.023

Cheyne, D., Bakhtazad, L. & Gaetz, W. (2008) Spatiotemporal mapping of cortical activity accompanying voluntary movements using an event-related beamforming approach. *NeuroImage*, 39(1), 19–29. doi:10.1016/j.neuroimage.2007.08.030.
URL https://doi.org/10.1016/j.neuroimage.2007.08.030

Cirelli, L.K., Jurewicz, Z.B. & Trehub, S.E. (2019) Effects of maternal singing style on mother–infant arousal and behavior. *Journal of Cognitive Neuroscience*, 32, 1213–1220.
URL https://doi.org/10.1162/jocn_a_01402

Clauset, A., Shalizi, C.R. & Newman, M.E.J. (2009) Power-law distributions in empirical data. *SIAM Review*, 51, 661–703.
URL https://doi.org/10.1137/070710111

Cuevas, A. & Fraiman, R. (2009) On depth measures and dual statistics. a methodology for dealing with general data. *Journal of Multivariate Analysis*, 100(4), 753–766.
URL https://doi.org/10.1016/j.jmva.2008.08.002

Daffertshofer, A., Ton, R., Kringelbach, M.L., Woolrich, M.W. & Deco, G. (2018) Distinct criticality of phase and amplitude dynamics in the resting brain. *NeuroImage*, 180, 442–447.
URL https://doi.org/10.1016/j.neuroimage.2018.03.002

Dahl, M.J., Mather, M. & Werkle-Bergner, M. (2022) Noradrenergic modulation of rhythmic neural activity shapes selective attention. *Trends in Cognitive Sciences*, 26(1), 38–52.
URL https://doi.org/10.1016/j.tics.2021.10.009

Deco, G., Garcia, S.L., Perl, Y.S., Sporns, O. & Kringelbach, M.L. (2023) The effect of turbulence in brain dynamics information transfer measured with magnetoencephalography. *Communications Physics*, 6, 1–8.
URL https://doi.org/10.1038/s42005-023-01192-2

Deco, G. & Kringelbach, M.L. (2020) Turbulent-like dynamics in the human brain. *Cell Rep.*, 33(10), 108471.
URL https://doi.org/10.1016/j.celrep.2020.108471

Deco, G., Perl, Y.S., Jerotic, K., Escrichs, A. & Kringelbach, M.L. (2025) Turbulence as a framework for brain dynamics in health and disease. *Neuroscience & Biobehavioral Reviews*, 169, 105988.
URL https://doi.org/10.1016/j.neubiorev.2024.105988

Ehinger, B.V., Fischer, P., Gert, A.L., Kaufhold, L., Weber, F., Pipa, G. et al. (2014) Kinesthetic and vestibular information modulate alpha activity during spatial navigation: a mobile eeg study. *Frontiers in Human Neuroscience*, 8, 71.
URL https://doi.org/10.3389/fnhum.2014.00071

Epifanio, I. & Ventura-Campos, N. (2014) Hippocampal shape analysis in alzheimer's disease using functional data analysis. *Statistics in Medicine*, 33(5), 867–880.
URL https://doi.org/10.1002/sim.5968

Escrichs, A., Perl, Y.S., Uribe, C., Camara, E., Türker, B., Pyatigorskaya, N. et al. (2022) Unifying turbulent dynamics framework distinguishes different brain states. *Communications Biology*, 5, 638.
URL https://doi.org/10.1038/s42003-022-03576-6

Fayomi, A., Pantazis, Y., Tsagris, M. & Wood, A.T.A. (2022) Cauchy robust principal component analysis with applications to high-dimensional data sets. *Statistics and Computing*, 34, 1–14.
URL https://doi.org/10.1007/s11222-023-10328-x

Fitch, W.T. (2005) The evolution of music in comparative perspective. *Annals of the New York Academy of Sciences*, 1060, 29–49.
URL https://doi.org/10.1196/annals.1360.004

Fourcade, A., Klotzsche, F., Hofmann, S.M., Mariola, A., Nikulin, V.V., Villringer, A. et al. (2024) Linking brain–heart interactions to emotional arousal in immersive virtual reality. *Psychophysiology*, 61(12), e14696.
URL https://doi.org/10.1111/psyp.14696

Foxe, J.J. & Snyder, A.C. (2011) The role of alpha-band brain oscillations as a sensory suppression mechanism during selective attention. *Frontiers in Psychology*, 2.
URL https://doi.org/10.3389/fpsyg.2011.00154

Gao, S., Proekt, A., Proekt, A., Renier, N., Calderon, D.P., Calderon, D.P. et al. (2019) Activating an anterior nucleus gigantocellularis subpopulation triggers emergence from pharmacologically-induced coma in rodents. *Nature Communications*, 10, 2897.
URL https://doi.org/10.1038/s41467-019-10797-7

Genzer, S., Ong, D.C., Zaki, J. & Perry, A. (2018) Mu rhythm suppression over sensorimotor regions is associated with greater empathic accuracy. *Social Cognitive and Affective Neuroscience*, 17(19), 788–801.
URL https://doi.org/10.1093/scan/nsac011

Gordon, E.M., Chauvin, R.J., Van, A.N., Rajesh, A., Nielsen, A., Newbold, D.J. et al. (2023) A somato-cognitive action network alternates with effector regions in motor cortex. *Nature*, 617(7960), 351–359.
URL https://doi.org/10.1038/s41586-023-05964-2

Gray, P.M., Krause, B.L., Atema, J., Payne, R.L., Krumhansl, C. & Baptista, L.F. (2001) The music of nature and the nature of music. *Science*, 291, 52–54.

Gritton, H., Booth, V. & Howe, W. (2024) Special issue on cholinergic signalling. *European Journal of Neuroscience*, 59(9), 2131–2137.
URL https://doi.org/10.1111/ejn.16369

Haegens, S., Nácher, V., Luna, R., Romo, R. & Jensen, O. (2011) α-oscillations in the monkey sensorimotor network influence discrimination performance by rhythmical inhibition of neuronal spiking. *Proceedings of the National Academy of Sciences*, 108, 19377–19382.
URL https://doi.org/10.1073/pnas.1117190108

Halgren, M., Ulbert, I., Bastuji, H., Fabó, D., Erőss, L., Rey, M. et al. (2019) The generation and propagation of the human alpha rhythm. *Proceedings of the National Academy of Sciences*, 116(47), 23772–23782.
URL https://doi.org/10.1073/pnas.1913092116

Haufe, S., Dähne, S. & Nikulin, V.V. (2014) Dimensionality reduction for the analysis of brain oscillations. *NeuroImage*, 101, 583–597.
URL https://doi.org/10.1016/j.neuroimage.2014.06.073

Herman, L.M. (2017) The multiple functions of male song within the humpback whale (megaptera novaeangliae) mating system: review, evaluation, and synthesis. *Biological Reviews*, 92.
URL https://doi.org/10.1111/brv.12309

Hofmann, S.M., Klotzsche, F., Mariola, A., Nikulin, V.V., Villringer, A. & Gaebler, M. (2020) Decoding subjective emotional arousal from EEG during an immersive virtual reality experience. *eLife*, 10, e64812.
URL https://doi.org/10.7554/eLife.64812

Holstege, G., Bandler, R. & Saper, C. (1996) *The Emotional Motor System*. Progress in Brain Research. : Elsevier.

Holstege, G. & Subramanian, H.H. (2015) Two different motor systems are needed to generate human speech. *Journal of Comparative Neurology*, 524, 1558–1577.
URL https://doi.org/10.1002/cne.23898

Hudson, A.E., Calderon, D.P., Pfaff, D.W. & Proekt, A. (2014) Recovery of consciousness is mediated by a network of discrete metastable activity states. *Proceedings of the National Academy of Sciences*, 111, 9283–9288.
URL https://doi.org/10.1073/pnas.1408296111

Itoh, K., Konoike, N., Nejime, M., Iwaoki, H., Igarashi, H., Hirata, S. et al. (2022) Cerebral cortical processing time is elongated in human brain evolution. *Scientific Reports*, 12(1).
URL https://doi.org/10.1038/s41598-022-05053-w

Iwasaki, M., Poulsen, T.M., Oka, K. & Hessler, N.A. (2013) Singing-related activity in anterior forebrain of male zebra finches reflects courtship motivation for target females. *PLoS ONE*, 8.
URL https://doi.org/10.1111/brv.12309

Jaffe, P.I. & Brainard, M.S. (2020) Acetylcholine acts on songbird premotor circuitry to invigorate vocal output. *eLife*, 9, e53288.
URL https://doi.org/10.7554/eLife.53288

Jonmohamadi, Y. & Muthukumaraswamy, S.D. (2017) Multi-band component analysis for EEG artifact removal and source reconstruction with application to gamma-band activity. *Biomedical Physics & Engineering Express*, 4, 035007.
URL https://doi.org/10.1088/2057-1976/aab0ce

Jover, M., Cellier, M. & Scola, C. (2019) Infants' motor activity during a mother–infant interaction alternating silent and singing phases. *Journal of Motor Learning and Development*, 7, 426–436.
URL https://doi.org/10.1123/jmld.2019-0001

Jürgens, U. (2009) The neural control of vocalization in mammals: a review. *Journal of voice: official journal of the Voice Foundation*, 23(1), 1–10.
URL https://doi.org/10.1016/j.jvoice.2007.07.005

Kaiser, J.F. (1990) On a simple algorithm to calculate the 'energy' of a signal. *International Conference on Acoustics, Speech, and Signal Processing*, 1, 381–384.
URL https://doi.org/10.1109/ICASSP.1990.115702

Kilinc, M., Calderon, D.P., Tabansky, I., Martin, E.M. & Pfaff, D.W. (2023) *Elementary Central Nervous System Arousal*. New York, NY: Springer New York.

Kim, J.H., Kim, D.W. & Im, C.H. (2017) Brain areas responsible for vigilance: An EEG source imaging study. *Brain Topography*, 30, 343–351.
URL https://doi.org/10.1007/s10548-016-0540-0

Kleber, B., Veit, R., Birbaumer, N., Gruzelier, J. & Lotze, M. (2010) The brain of opera singers: experience-dependent changes in functional activation. *Cerebral Cortex*, 20(5), 1144–1152.
URL https://doi.org/10.1093/cercor/bhp177

Kleber, B., Zeitouni, A., Friberg, A. & Zatorre, R.J. (2013) Experience-dependent modulation of feedback integration during singing: Role of the right anterior insula. *The Journal of Neuroscience*, 33, 6070–6080.
URL https://doi.org/10.1523/JNEUROSCI.4418-12.2013

Koelsch, S., Fritz, T., v. Cramon, D.Y., Müller, K. & Friederici, A.D. (2006) Investigating emotion with music: An fMRI study. *Human Brain Mapping*, 27.
URL https://doi.org/10.1002/hbm.20180.

Koner, S. & Staicu, A.M. (2023) Second-generation functional data. *Annual Review of Statistics and Its Application*, 10(1), 547–572.
URL https://doi.org/10.1146/annurev-statistics-032921-033726

Koo, T.K. & Li, M.Y. (2016) A guideline of selecting and reporting intraclass correlation coefficients for reliability research. *Journal of Chiropractic Medicine*, 15 2, 155–63.
URL https://doi.org/0.1016/j.jcm.2016.02.012

Leman, M. (2008) *Embodied music cognition and mediation technology*. : MIT Press.

Lense, M.D., Shultz, S., Astésano, C. & Jones, W. (2022) Music of infant-directed singing entrains infants' social visual behavior. *Proceedings of the National Academy of Sciences*, 119, e2116967119. URL https://doi.org/10.1073/pnas.2116967119

Lévêque, Y. & Schön, D. (2015) Modulation of the motor cortex during singing-voice perception. *Neuropsychologia*, 70, 58–63. URL https://doi.org/10.1016/j.neuropsychologia.2015.02.012

Liebisch, A.P., Eggert, T., Shindy, A., Valentini, E., Irving, S., Stankewitz, A. et al. (2020) A novel tool for the removal of muscle artefacts from eeg: Improving data quality in the gamma frequency range. *Journal of Neuroscience Methods*, 358, 109217. URL https://doi.org/10.1016/j.jneumeth.2021.109217.

Lohani, S., Moberly, A.H., Benisty, H., Landa, B., Jing, M., Li, Y. et al. (2022) Spatiotemporally heterogeneous coordination of cholinergic and neocortical activity. *Nature Neuroscience*, 25, 1706–1713. URL https://doi.org/10.1038/s41593-022-01202-6

Martin, E.M. & Pfaff, D.W. (2013) *Elementary CNS Arousal*. New York, NY: Springer New York.

Moura, N., Fonseca, P., Vilas-Boas, J.P. & Serra, S. (2024) Increased body movement equals better performance? not always! musical style determines motion degree perceived as optimal in music performance. *Psychological Research*, 88, 1314–1330. URL https://doi.org/10.1007/s00426-024-01928-x

Muthukumaraswamy, S.D. (2010) Functional properties of human primary motor cortex gamma oscillations. *Journal of Neurophysiology*, 104(5), 2873–2885. doi:10.1152/jn.00607.2010. URL https://doi.org/10.1152/jn.00607.2010

Muthukumaraswamy, S.D. (2013) High-frequency brain activity and muscle artifacts in MEG/EEG: a review and recommendations. *Frontiers in Human Neuroscience*, 7(138). URL https://doi.org/10.3389/fnhum.2013.00138

Nair, A., Teo, Y.Y., Augustine, G.J. & Graf, M. (2023) A functional logic for neurotransmitter corelease in the cholinergic forebrain pathway. *Proceedings of the National Academy of Sciences*, 120(28), e2218830120. URL https://doi.org/10.1073/pnas.2218830120

Nikulin, V.V., Nolte, G. & Curio, G. (2011) A novel method for reliable and fast extraction of neuronal EEG/MEG oscillations on the basis of spatio-spectral decomposition. *NeuroImage*, 55, 1528–1535. URL https://doi.org/10.1016/j.neuroimage.2011.01.057

Noei, S., Zouridis, I.S., Logothetis, N.K., Panzeri, S. & Totah, N.K. (2022) Distinct ensembles in the noradrenergic locus coeruleus are associated with diverse cortical states. *Proceedings of the National Academy of Sciences*, 119(18), e2116507119. URL https://doi.org/10.1073/pnas.2116507119

Norman-Haignere, S.V., Feather, J., Boebinger, D., Brunner, P., Ritaccio, A.L., McDermott, J.H. et al. (2022) A neural population selective for song in human auditory cortex. *Current Biology*, 32, 1470–1484.e12. URL https://doi.org/10.1016/j.cub.2022.01.069

Owren, M.J., Amoss, R.T. & Rendall, D. (2011) Two organizing principles of vocal production: Implications for nonhuman and human primates. *American Journal of Primatology*, 73(6), 530–544. URL https://doi.org/10.1002/ajp.20913

Percival, D.B. & Mofjeld, H.O. (1997) Analysis of subtidal coastal sea level fluctuations using wavelets. *Journal of the American Statistical Association*, 92, 868–880. URL https://doi.org/10.1080/01621459.1997.10474042

Pfaff, D.W. (2009) *Brain Arousal and Information Theory: Neural and Genetic Mechanisms*. : Harvard University Press.

Pfaff, D.W., Martin, E.M. & Faber, D.S. (2012) Origins of arousal: roles for medullary reticular neurons. *Trends Neuroscience*, 35, 468–476. URL https://doi.org/10.1016/j.tins.2012.04.008

Pozeg, P., Palluel, E., Ronchi, R., Solcà, M., Al-Khodairy, A., Jordan, X. et al. (2017) Virtual reality improves embodiment and neuropathic pain caused by spinal cord injury. *Neurology*, 89, 1894–1903. URL https://doi.org/10.1212/WNL.0000000000004585

Proekt, A., Banavar, J.R., Maritan, A. & Pfaff, D.W. (2012) Scale invariance in the dynamics of spontaneous behavior. *Proceedings of the National Academy of Sciences*, 109, 10564–10569. URL https://doi.org/10.1073/pnas.1206894109

Quinkert, A.W., Vimal, V.P., Weil, Z.M., Reeke, G.N., Schiff, N.D., Banavar, J.R. et al. (2011) Quantitative descriptions of generalized arousal, an elementary function of the vertebrate brain. *Proceedings of the National Academy of Sciences*, 108, 15617–15623. URL https://doi.org/10.1073/pnas.1101894108

R Core Team (2021) *R: A Language and Environment for Statistical Computing*. R Foundation for Statistical Computing, Vienna, Austria. URL https://www.R-project.org/

Reimer, J., McGinley, M.J., Liu, Y., Rodenkirch, C., Wang, Q., McCormick, D.A. et al. (2016) Pupil fluctuations track rapid changes in adrenergic and cholinergic activity in cortex. *Nature Communications*, 7, 13289. URL https://doi.org/10.1038/ncomms13289

Ribeiro, A.B., Gabriel, R., Garcia, B., Cuccio, C., Aqeel, W., Moreno, A. et al. (2022) Temporal relations between peripheral and central arousals in good and poor sleepers. *Proceedings of the National Academy of Sciences*, 119. URL https://doi.org/10.1073/pnas.2201143119

Rosa, M.D., Sangalli, L.M. & Vantini, S. (2014) Principal differential analysis of the aneurisk65 data set. *Advances in Data Analysis and Classification*, 8, 287–302. URL https://doi.org/10.1007/s11634-014-0175-5

Ross, J.M., Comstock, D.C., Iversen, J.R., Makeig, S. & Balasubramaniam, R. (2021) Cortical mu rhythms during action and passive music listening. *Journal of Neurophysiology*,. URL https://doi.org/10.1152/jn.00346.2021

Rousseeuw, P.J. & van Driessen, K. (1999) A fast algorithm for the minimum covariance determinant estimator. *Technometrics*, 41, 212–223. URL https://doi.org/10.2307/1270566

Roy, S., Banerjee, A., Roy, C., Nag, S., Sanyal, S., Sengupta, R. et al. (2021) Brain response to color stimuli: an EEG study with nonlinear approach. *Cognitive Neurodynamics*, 15(6), 1023–1053. URL https://doi.org/10.1007/s11571-021-09692-z

Sasaki, A., Sotnikova, T.D., Gainetdinov, R.R. & Jarvis, E.D. (2006) Social context-dependent singing-regulated dopamine. *The Journal of Neuroscience*, 26, 9010–9014. URL https://doi.org/10.1523/JNEUROSCI.1335-06.2006

Schaworonkow, N. & Nikulin, V.V. (2018) Spatial neuronal synchronization and the waveform of oscillations: Implications for EEG and MEG. *PLoS Computational Biology*, 15. URL https://doi.org/10.1371/journal.pcbi.1007055

Siman-Tov, T., Gordon, C.R., Avisdris, N., Shany, O., Lerner, A., Shuster, O. et al. (2022) The rediscovered motor-related area 55b emerges as a core hub of music perception. *Communications Biology*, 5, 1104. URL https://doi.org/10.1038/s42003-022-04009-0

Siqi-Liu, A., Harris, A., Atkinson, A.P. & Reed, C.L. (2018) Dissociable processing of emotional and neutral body movements revealed by μ-alpha and beta rhythms. *Social Cognitive and Affective Neuroscience*, 13(12), 1269–1279. URL https://doi.org/10.1093/scan/nsy094

Spiech, C., Danielsen, A., Laeng, B. & Endestad, T. (2024) Oscillatory attention in groove. *Cortex*, 174, 137–148. URL https://doi.org/10.1016/j.cortex.2024.02.013

Staib, M. & Frühholz, S. (2021) Cortical voice processing is grounded in elementary sound analyses for vocalization relevant sound patterns. *Progress in Neurobiology*, 200.

URL https://doi.org/10.1016/j.pneurobio.2020.101982
Stitt, I., Zhou, Z.C., Radtke-Schuller, S. & Fröhlich, F. (2018) Arousal dependent modulation of thalamo-cortical functional interaction. *Nature Communications*, 9(1), 2455.
URL https://doi.org/10.1038/s41467-018-04785-6
Vidal, M. & Aguilera, A.M. (2025) Wavelet thresholding on independent subspace factorizations of spatially indexed wide functional data for robust estimation of cortical activity. *Mathematics and Computers in Simulation*, 232, 346–361.
URL https://doi.org/10.1016/j.matcom.2025.01.012
Vidal, M. & Aguilera, M. (2023) Novel whitening approaches in functional settings. *Stat*, 12(1), e516.
URL https://doi.org/10.1002/sta4.516
Vidal, M., Leman, M. & Aguilera, A.M. (2025) Functional independent component analysis by choice of norm: a framework for near-perfect classification. *Advances in Data Analysis and Classification*,.
URL https://doi.org/10.1007/s11634-024-00622-5
Vidal, M., Onderdijk, E., K., Aguilera, A.M., Six, J., Maes, P.J. et al. (2024) Cholinergic-related pupil activity reflects level of emotionality during motor performance. *European Journal of Neuroscience*, 59(9), 2193–2207.
URL https://doi.org/10.1111/ejn.15998
Vidal, M., Rosso, M. & Aguilera, A.M. (2021) Bi-smoothed functional independent component analysis of EEG data. *Mathematics*, 9, 1243.
URL https://doi.org/10.3390/math9111243
Vinck, M., Uran, C., Dowdall, J.R., Rummell, B. & Canales-Johnson, A. (2025) Large-scale interactions in predictive processing: oscillatory versus transient dynamics. *Trends in Cognitive Sciences*, 29(2), 133–146.
URL https://doi.org/10.1016/j.tics.2024.09.013
Wang, Y., Siu, T.S.C. & Cheung, H. (2023) Effect of music emotion on mu and beta oscillations. *Psychology of Music*, 51(5), 1489–1500.
URL https://doi.org/10.1177/03057356221145960
Washburn, A., Román, I., Huberth, M., Gang, N., Dauer, T., Reid, W. et al. (2019) Musical role asymmetries in piano duet performance influence alpha-band neural oscillation and behavioral synchronization. *Frontiers in Neuroscience*, 13, 1088.
URL https://doi.org/10.3389/fnins.2019.01088
Weber, D., Hertweck, S., Alwanni, H., Fiederer, L., Wang, X., Unruh, F. et al. (2021) A structured approach to test the signal quality of electroencephalography measurements during use of head-mounted displays for virtual reality applications. *Frontiers in Neuroscience*, 15, 733673.
URL https://doi.org/10.3389/fnins.2021.733673
Weinstein, D., Launay, J., Pearce, E., Dunbar, R.I.M. & Stewart, L. (2016) Singing and social bonding: changes in connectivity and pain threshold as a function of group size. *Evolution and Human Behavior*, 37, 152–158.
URL https://doi.org/10.1016/j.evolhumbehav.2015.10.002
Whitham, E.M., Pope, K.J., Fitzgibbon, S.P., Lewis, T., Clark, C.R., Loveless, S. et al. (2007) Scalp electrical recording during paralysis: quantitative evidence that eeg frequencies above 20 hz are contaminated by emg. *Clinical Neurophysiology*, 118(8), 1877–1888.
URL https://doi.org/10.1016/j.clinph.2007.04.027
Xu, G., Mihaylova, T., Li, D., Tian, F., Farrehi, P.M., Parent, J.M. et al. (2023) Surge of neurophysiological coupling and connectivity of gamma oscillations in the dying human brain. *Proceedings of the National Academy of Sciences*, 120.
URL https://doi.org/10.1073/pnas.2216268120
Yang, Y., Cui, X., Gong, H., Liu, Z., Wang, H. & Yan, B. (2020) High gamma band eeg closely related to emotion: Evidence from functional network. *Frontiers in Human Neuroscience*, 14, 89.
URL https://doi.org/10.3389/fnhum.2020.00089
Zarate, J.M. (2013) The neural control of singing. *Frontiers in Human Neuroscience*, 7(237).
URL https://doi.org/10.3389/fnhum.2013.00237
Zima, M., Zima, M., Tichavský, P., Paul, K. & Krajca, V. (2012) Robust removal of short-duration artifacts in long neonatal EEG recordings using wavelet-enhanced ICA and adaptive combining of tentative reconstructions. *Physiological Measurement*, 33, N39–N49.
URL https://doi.org/10.1088/0967-3334/33/8/N39

SUPPORTING INFORMATION

Anonymized data has been deposited at https://osf.io/c3t78
